# Genetic Variants of Matrix Metalloproteinase and Sepsis: The Need Speed Study

**DOI:** 10.3390/biom12020279

**Published:** 2022-02-09

**Authors:** Nicola Fiotti, Filippo Mearelli, Filippo Giorgio Di Girolamo, Luigi Mario Castello, Alessio Nunnari, Salvatore Di Somma, Enrico Lupia, Efrem Colonetti, Maria Lorenza Muiesan, Giuseppe Montrucchio, Carlo Giansante, Gian Carlo Avanzi, Gianni Biolo

**Affiliations:** 1 Unit of Internal Medicine, Department of Medical Surgical and Health Sciences, University of Trieste, 34149 Trieste, Italy; filippome@libero.it (F.M.); fgdigirolamo@units.it (F.G.D.G.); alessionunnari@gmail.com (A.N.); giansant@units.it (C.G.); biolo@units.it (G.B.); 2 SC Assistenza Farmaceutica, Cattinara Hospital, Azienda Sanitaria Universitaria Integrata di Trieste, 34149 Trieste, Italy; 3 Department of Translational Medicine, Università del Piemonte Orientale, Internal Medicine, A.O. “Santi Antonio e Biagio e Cesare Arrigo”, 15121 Alessandria, Italy; luigi.castello@med.uniupo.it; 4 Unit of Emergency Medicine, Department of Medical Surgery Sciences and Translational Medicine, University “Sapienza” of Rome, 00185 Roma, Italy; salvatore.disomma@uniroma1.it; 5 Unit of Emergency Medicine, Department of Medical Sciences, University of Turin, 10124 Torino, Italy; enrico.lupia@unito.it (E.L.); giuseppe.montrucchio@unito.it (G.M.); 6 Unit of Internal Medicine, Department of Clinical and Experimental Sciences, University of Brescia, 25121 Brescia, Italy; colonetti@gmail.com (E.C.); marialorenza.muiesan@unibs.it (M.L.M.); 7 Department of Translational Medicine, Università del Piemonte Orientale, Via Solaroli 17, 28100 Novara, Italy; giancarlo.avanzi@uniupo.it

**Keywords:** matrix metalloproteinases polymorphism, sepsis, SIRS, fever, hyperthermia

## Abstract

Many causal mechanisms in sepsis susceptibility are largely unknown and the functional genetic polymorphisms (GP) of matrix metalloproteinases (MMPs) and their natural tissue inhibitor of MMPs (TIMP1) could play a role in its development. GPs of MMPs and TIMP (namely MMP-1 rs1799750, MMP-3 rs3025058, MMP-8 rs11225395, MMP-9 rs2234681, and TIMP-1 rs4898) have been compared in 1058 patients with suspected sepsis to assess the association with susceptibility and etiology of sepsis. Prevalence of MMP8 rs11225395 G/G genotype was higher in sepsis patients than in those with non-infective Systemic Inflammatory Reaction Syndrome (35.6 vs. 26%, hazard ratio, HR 1.56, 95% C.I. 1.04–2.42, *p* = 0.032). G/G patients developed less hyperthermia (*p* = 0.041), even after stratification for disease severity (*p* = 0.003). Patients carrying the 6A allele in MMP3 rs3025058 had a higher probability of microbiologically-proven sepsis (HR 1.4. 95%C.I. 1.01–1.94, *p* = *0*.044), particularly when due to virus (H.R. 2.14, 95% C.I. 1.06–4.31, *p* = 0.046), while MMP-1 G/G genotype patients carried a higher risk for intracellular bacteria (Chlamydia, Mycoplasma, and Legionella, H.R. 6.46, 95% C.I. 1.58–26.41, *p* = 0.003). Neither severity of sepsis at presentation, nor 30-day mortality were influenced by the investigated variants or their haplotype. MMP8 rs11225395 G/G carriers have lower temperature at presentation and a more than 50% increased susceptibility to sepsis. Among patients with sepsis, carriers of MMP1 rs1799750 G/G have an increased susceptibility for intracellular pathogen infections, while virus serology is more often positive in those with the MMP3 rs3025058 A/A genotype.

## 1. Introduction

Sepsis, the invasion of microorganisms inducing a generalized inflammatory response, is a major cause of death and permanent disability in Western countries and worldwide [1]. Its occurrence and outcomes reflect the balance between, on one side, the invading capacities of the pathogen, and on the other, mechanical and immune (innate and acquired) mechanisms. Both defense mechanisms have key players in matrix metalloproteinases (MMPs) and their inhibitors (tissue inhibitors of matrix metalloproteinases, TIMPs) [2]. MMPs are calcium and zinc-dependent proteolytic enzymes that can be membrane-bound or soluble (secreted), which are able to degrade all components of the extracellular matrix, thereby favoring cell migration within tissues and enhancing the availability of growth factors bound to the matrix. Over the years, many non-structural and intracellular targets of MMPs have been identified, thus expanding their potential role in immune modulation [3,4,5] and other cellular functions [6].

MMP activities are tightly regulated through epigenetic, transcriptional, and post-transcriptional modulation of gene expression, proteolytic activation, post-translational modifications, and extracellular inhibition [7]. Among these, functional polymorphisms influence gene expression and activity and might therefore control the pathogen invasion process, thus influencing susceptibility, clinical presentation, and likely the outcomes of sepsis. Inhibition and clearance of these MMPs are performed by specific inhibitors (tissue inhibitor of metalloprotease, TIMP 1–4), which, in turn, undergo regulation of its expression by genetic polymorphisms. At present, knowledge on the role of MMP/TIMP polymorphisms has been derived from small studies mainly focused on the prognostic value of these variants. Nonetheless, their differential expression might identify specific pathways for the susceptibility and presentation of sepsis compared to other inflammatory non-septic conditions.

The role of MMPs in sepsis has already been addressed in experimental and clinical conditions, although clinical investigation has mainly focused on plasma levels [8,9,10]. A major limitation of this type of approach is that plasma MMP or TIMP concentration fluctuates in sepsis according to the stage of the disease, and each MMP might have different timing in appearance, some being immediately released by activated neutrophils (e.g., MMP-8 and MMP-9) [11] while others are expressed and released by inflamed tissues over a different time period [12]. In this context, functional genetic variants would enable us to estimate the in vivo effects of a different expression of MMP or TIMP within tissues free from the timing of the sample collection. In the choice of MMP/TIMP and their genetic variants, we started from MMPs with increased/decreased expression (or plasma levels) during sepsis [13], with functional genetic variants and adequate allelic frequency and, when available, connected with expression pathways known to be activated during sepsis, (e.g., *ets* or NF-kB) [14,15,16].

In the present study, we investigated the association of five functional polymorphisms of MMP-1 rs1799750, MMP-3 rs3025058, MMP-8 rs11225395, MMP-9 rs2234681, and TIMP-1 rs4898 with clinical presentation of Systemic Inflammatory Response Syndrome (SIRS), sepsis susceptibility, etiology, and survival in a cohort of patients from the Need Speed study.

## 2. Materials and Methods

### 2.1. Patients and Study Design

The population study was selected from the cohort of the Need Speed study, an Italian multicenter study on the diagnosis of sepsis, the study design and results of which have already been published [17,18]. Briefly, patients admitted to ER or medical wards with at least two SIRS criteria [19] and suspicion of sepsis were enrolled. Exclusion criteria were minor age and refusal to participate to the study. Each patient or legal representative signed a written informed consent form to participate in the study. Blood samples were taken at admission and diagnostic workout carried out according to the principles of good clinical practice and blinding of the results of the research. A flowchart of the study is shown in Supplementary Figure S1. Comorbidities, severity of condition, final diagnosis, and 30-day survival were considered. Each researcher in charge of the patient conveyed the final diagnosis and the development of complications. In order to balance different inter-researcher interpretations, an independent committee (i.e., colleagues involved in the study but not in clinical workout of the specific patient) validated the diagnosis and complications. Disagreements between doctors in charge and the committee were discussed and a final diagnosis was formulated for the study analysis. All of the above evaluations (by the researcher and by the committee) were blinded to the results of the research and genetic test. The final diagnoses of each patient were grouped into three categories: definite sepsis; non-infective SIRS, when a definite diagnosis alternative to sepsis was posed; and debatable, when no definite or exclusive evidence of sepsis or Ni-SIRS was reached. In turn, sepsis patients were divided in either microbiologically proven, when clinical course plus culture test (from blood, urine, sputum, pleural liquid, cerebrospinal fluid, abscess material), serology, PCR based amplification of pathogen genetic material, immunofluorescence-based antigen detection, identified one or more microorganisms likely to be causative of sepsis, or clinical sepsis when imaging and/or clinical course were suggestive but with negative or uncertain culture test. In the case of more pathogens observed in the same patients, only those microorganisms alleged to contribute to sepsis were considered for further analysis.

All patients with a definite diagnosis were included to assess susceptibility to sepsis, while only those with culture or serology positive workout were investigated for the association of MMP variants with susceptibility to a specific pathogen. Patient outcome considered for association analysis were 30-day survival. The present sub-analysis was conducted on all patients providing the genetic material.

The study complies with the Declaration of Helsinki and was approved by the Ethical Committee of each institution participating in the study.

### 2.2. DNA Extraction and Polymorphism Analysis

DNA was extracted from a venous blood sample with a suitable extraction kit on Maxwell 16™ (both products from Promega Italia) according to the manufacturer’s instructions. For MMP-1 rs1799750 and MMP-3 rs3025058, which are ins/del variants and MMP-9 rs2234681, which is a variable number of tandem repeats (VNTR, 13–26 CA repeats around −90 from transcription start), the amplicon size (informative of genotype) was assessed through capillary electrophoresis according to the methods already published [20]. The repeats in the MMP9 rs2234681 allele were grouped in those with 21 or less repeats or 22 or more, according to a previous work [21]. For MMP-8 rs11225395 and TIMP-1 rs 4898, which are substitution variants, commercial kits (Taqman technology, catalog number C 1,366,493 20 and C 11,175,659 10, respectively, from Lifetechnologies) were assessed in Real-Time Biorad according to the manufacturer’s instructions.

Patients were genotyped by expert personnel at the University of Trieste blinded to any clinical data except for gender during (X-linked) TIMP-1 genotyping. Results of such variants were analyzed and reported as carriers (or not) of the T(A) allele, independent of the gender of the carrier, consistent with a previous paper [22].

Hardy–Weinberg (HW) equilibrium and Linkage disequilibrium (for MMP-1, MMP-8, and MMP-3, D′) as well as haplotype analysis were determined using the online calculator available at https://www.snpstats.net/ (accessed on 20 December 2021) [23]. In MMP-9 rs2234681, alleles were considered as short and long and the HW calculated accordingly [24]. For the X-linked TIMP-1 gene, HW was assessed for female patients and allelic frequencies between the two sexes were compared.

Genetic material was handled according to the Italian guidelines for the treatment of genetic material (General Authorization No. 8/2013 for the Processing of Genetic Data, from the “Garante per la protezione dei dati personali”, accessible at http://www.garanteprivacy.it/web/guest/home/docweb/-/docweb-display/docweb/2818993 (accessed on 20 December 2021).

### 2.3. Statistical Analysis

Categorical or continuous variable data are reported as the number of observations and prevalence or median and interquartile (IQR) values, respectively. Associations among categorical variables were investigated with the chi-square (χ^2^) test, hazard ratio and 95%CI. For continuous variables, the skewed data distribution of some of these called for non-parametric statistics. Consequently, all comparisons of the continuous variables among categorical variables were investigated using the Mann–Whitney or Kruskal–Wallis test, and linear correlation was analyzed by the Spearman’s Rho ranking test. Survival analysis was performed with the Kaplan–Maier test and a comparison within the categorical variables was obtained through the logarithmic rank test (Mantel Cox). Unconditional binary logistic regression analysis was used to examine the constructed 95% confidence intervals (95% CIs) and hazard ratios (HRs), for genetic variants in the final diagnosis of sepsis together with variants used for previous work [17], or for specific diagnosis of viral or intracellular pathogen diagnosis for the codominant, dominant, recessive, and sovradominant models. Association of genetic variables was conducted assuming a co-dominant, dominant, recessive, or over-dominant model [25]. Haplotype analysis was conducted to investigate the interaction of polymorphisms in association with the clinical variable investigated. Comparison of genotypes and genotype/haplotype interaction were based on the comparison of HR and 95% CI.

A two tailed *p*-value less than 0.05 or HR 95%CI not encompassing the “1.0” value was considered statistically significant. All statistical analysis was conducted with SPSS 21.0 (Statistical Package for Social Sciences, SPSS Inc., Chicago, IL, USA).

## 3. Results

### 3.1. Population and MMP/TIMP Polymorphism

In total, 1058 patients were analyzed in the present sub-study. Among these, a definite diagnosis was reached in 939 patients: 127 non infective SIRS (Ni-SIRS) and 812 sepsis, leaving debatable diagnosis (SIRS cases that could not be robustly characterized as infected) in the remaining 119. Clinical and laboratory data were complete in more than 99% of cases. Genotyping was successful in 99.5% of patients overall with few patients not fully genotyped (namely 6, 7, 3, 3, and 8 patients for MMP-1, -3, -8, -9, and TIMP-1, respectively). HW equilibrium was maintained for MMP1, -3, and -9 (*p* = 0.99, 0.354, 0.181, respectively) and for TIMP-1 female patients, (*p* = 0.857, T allelic frequency of 0.57 and 0.566 in the female and male sex, respectively). HW equilibrium for MMP8 was not demonstrated (*p* = *0*.000029) with increased heterozygosity. Furthermore, HW equilibrium was reevaluated in patients with a definite diagnosis, with no remarkable differences from the whole population. Consistently, for MMP-8 rs11225395, HW was not confirmed in all groups of patients (*p* = 0.02, 0.0022, and 0.039 for non-infective SIRS, sepsis, and undefined patients, respectively). Linkage disequilibrium between MMP-1 rs1799750 and MMP-8 rs11225395 was demonstrated (D stat = 0.0241, D’ = 0.12 r = 0.0992 *p* =< 0.0001). No difference in genetic frequencies could be detected comparing the different centers contributing to the study (all *p* > 0.05). Patients’ general characteristics, SIRS criteria, clinical variables at presentation, and severity and comorbidity indexes are reported in Table 1 grouped according to the final diagnosis.

The association of MMP polymorphisms with features of presentation were examined independent of final diagnosis.

### 3.2. MMP/TIMP Genotype and Sepsis Susceptibility

**Figure 1:**
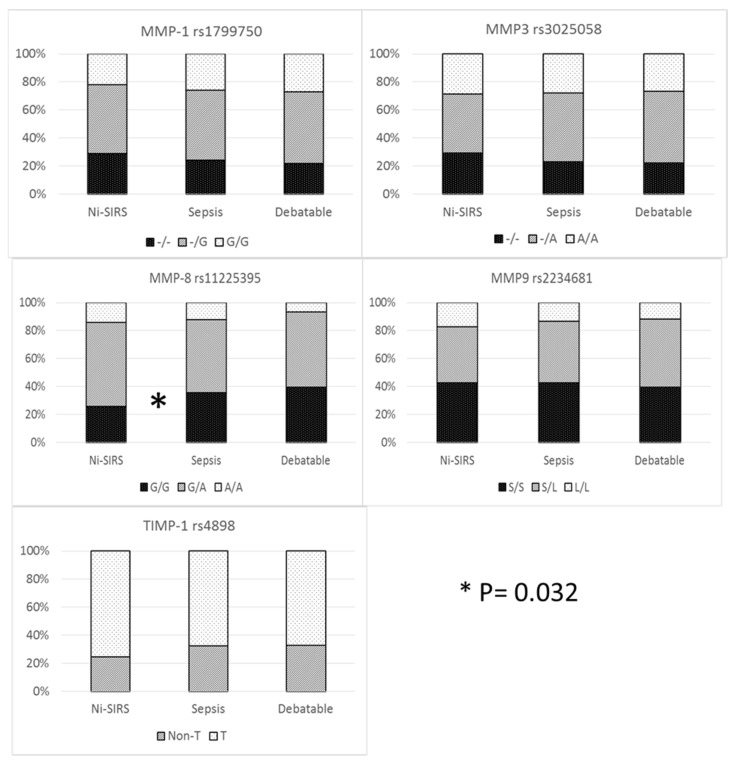
MMP/TIMP genetic variables and diagnosis.

The prevalence of different genotypes was compared only in patients with definite diagnosis (i.e., sepsis and non-infective SIRS). Allelic and genetic prevalence are reported in Figure 1 and Table S1, Supplementary Materials. Cross tabulation of genetic prevalence was marginally significant only for MMP-8 (codominant model), although MMP-8 G/G genotype (recessive model) was more prevalent in sepsis patients than in non-infective SIRS patients (35.6 vs. 26%) with an increase in risk of 58% (95%CI 1.04–2.42).

Haplotype analysis was conducted to observe additive diagnostic efficacy of MMP-8 rs11225395, MMP-9 rs2234681, and TIMP-1 rs4898 haplotype over the MMP-8 genotype. No advantage was demonstrated for haplotype analysis of MMP-8 and MMP-9 (global haplotype association *p* value = 0.24) and when TIMP-1 rs4898 was considered as a covariate (interaction *p* value = 0.2).

### 3.3. Clinical Presentation and MMP-8 rs11225395

Further investigation was carried out on MMP-8 polymorphism and its association with clinical presentation. SIRS criteria between Ni-SIRS and sepsis are summarized in Table 1 and their comparison according to MMP-8 genotype for Ni-SIRS and sepsis is reported in Table 2.

**Figure 2:**
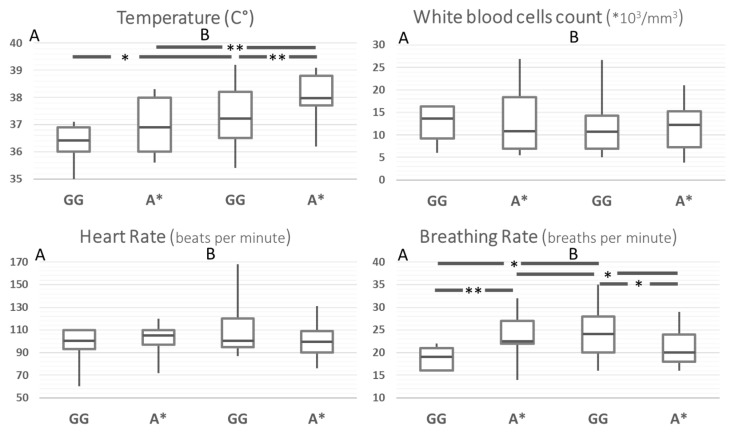
SIRS criteria according to MMP8 rs11225395 polymorphism in patients with Ni-SIRS (**A**) and sepsis (**B**) standardized for diabetes, age, and severity (SOFA score). ** = *p* < 0.01, * = *p* < 0.05.

In order to rule out the effect of possible confounders in the comparison between Ni-SIRS and septic patients, the same comparisons were carried out after stratification for age and disease severity (SOFA, Charlson, Apache II, and SAPS score). This yielded a group of 91 patients, whose results are reported in Figure 2 and Supplementary Table S2. After standardization, body temperature at admission was the only SIRS criteria remaining higher in septic patients and different according to MMP8 genotype. Compared to the G/G genotype, A carriers had an increased risk for hyperthermia across the whole group and in the stratified septic patients (HR 4.4, 95%CI 1.7–11.4, *p* = 0.0024 and 5.8, 95%CI 1.9–18.2, *p* = 0.0024, respectively). Breathing rate showed a statistically weak paradoxical pattern, particularly with a higher rate of A carriers in Ni-SIRS patients.

### 3.4. Severity and Diagnosis of Patients with Sepsis

Among the 812 sepsis patients with available genetic data, there was no association between MMP genetic variants and severity, prognosis, or type of diagnosis, the results of which are reported in Table S3, Supplementary Materials. Comorbidities, assessed with the Charlson Comorbidity Index and severity of condition measured with APACHE II, SAPS, and SOFA score did not differ, according to the genotype. The results are reported in Table S4.

### 3.5. Pathogens and Genetic Variants

A list of isolated microorganisms is reported in Table S5, Supplementary Materials. Within patients with microbiologically proven sepsis, an association between some pathogens and MMP genetic variants was occasionally found, although none could stand the statistical multiple comparisons correction (described in detail in the legend of Table S4, Supplementary Materials. Additionally, some statistical trends (i.e., *p* < 0.1 and > 0.05) could also be observed. Collectively, small intracellular bacteria and viruses did not reach statistical significance when analyzed individually, but when grouped, the association with MMP-1 rs1799750 and MMP3 rs3025058, respectively, became strong (*p* = 0.004 for both).

**Figure 3:**
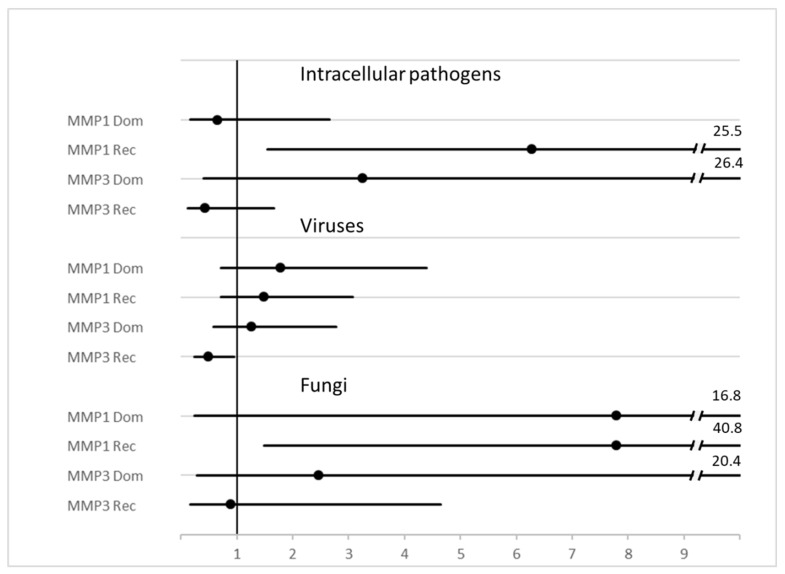
Forest plot of HR and 95%CI of MMP genotype variants and diagnosis of some classes of pathogens. Legend: MMP1 = rs1799750, MMP3 = rs3025058.

Evaluation of MMP polymorphisms have also been reported when grouping the pathogens according to Gram staining properties (Gram positive or negative), intracellular (atypical) bacteria, viruses, and fungi, amongst others, is reported in Table 3. Hazard ratios associated with diagnosis of a specific class of pathogens is reported as a forest plot in Figure 3. *p* = χ^2^ test for all diagnoses.

### 3.6. Binary Logistic Regression

To integrate the predictive role of MMP polymorphisms in the diagnosis of sepsis, binary logistic regression considered as the dependent variable the diagnosis of sepsis, sepsis due to virus, or intracellular pathogens and as independent variables, those available at admission (detailed in the Materials and Methods). The results are reported in Table 4.

Gram positive or negative bacteria as well as fungus sepsis did not show any association with MMP genetic variants as well as having a sepsis by more than one pathogen (data not shown).

## 4. Discussion

The present study reports the associations between MMP-8 rs11225395 G/G genotype and sepsis compared to patients without sepsis and, moreover, of the MMP-1 rs1799750 and MMP-3 rs3025058 genotypes with diagnosis of sepsis due to intracellular pathogens or virus, respectively.

### 4.1. MMP-8 and Sepsis

MMP-8 is mainly expressed in neutrophils or macrophages, while at later stages of acute lung injury, it can be released from mesenchymal cells in the airways [26]. MMP-8 is upregulated in sepsis [27], although laboratory models and molecular biology only show that its presence ensures neutrophil infiltration in response to lipopolysaccharide [28], likely through the degradation of LIX CXC chemokines [29]. This would limit lung inflammation and act as a key mediator in the regulation of innate immunity. Previous evidence that MMP-8 plays a role in sepsis is heterogeneous: in the cecal ligation perforation procedure, MMP-8 inhibition increases survival [30] and high plasma values predict sepsis compared with other patients admitted to ICU [10].

MMP-8 rs11225395, a genetic variant substitution at position −799, has two variants, with the A variant showing a higher mRNA expression [31,32]. The allelic frequency of the A variant in the general population ranges from 18 to 45%, according to the different populations studied, with the highest prevalence in the European and Mexican populations (available at https://www.snpedia.com/index.php/Rs11225395 (accessed on 3 February 2022). This variant has been previously associated with many different conditions such as cancer [33,34], aggressive periodontitis [35], recurrent pregnancy loss [36], and inflammation in atherosclerosis [32]. In all these studies, Hardy–Weinberg was maintained. Our first and somewhat surprising finding is that the HW equilibrium in our study was not maintained, and this independent of final diagnosis and recruiting center. In investigating the role of MMP-8 rs11225395 in sepsis, only one study has evaluated this variant, with negative results [10,37]. The relatively small size of the study and the different enrolling criteria might have hidden the association and the deviation from the HW equilibrium. Of note, MMP-1, -3, and -8 lie around 74 kb apart on the long arm of chromosome 11 and are in linkage disequilibrium. In our study, only rs11225395 showed deviance from the HW equilibrium, with a significant increase in heterozygosity. Classically, violation of HWE can be due to technical reasons or selection of the population. Among the former, possible causes are high minor allele frequency, low genotyping rate/success (typically <98%), or insertion/deletion variant [38]. As our genotyping rate was >99.5% (using a widely accepted technique) for a substitution variant, only the high prevalence of a minor allele could hypothetically explain the HW violation. The observation that in a sub-sample of 2196 variants with high (>98%) genotyping rate, no gain of heterozygosity was detected [38] further challenges this hypothesis. In conclusion, the most reliable reason for HW violation is patient selection. Indeed, enrolment in the Need Speed study was restricted to subjects developing no less than two signs/findings of systemic inflammation in response to infective or non-infective stimulus (SIRS criteria).

It was then important to detect which of the SIRS criteria could account for such a selection of the population leading to the violation of the HWE of rs11225395. To overcome the differences in the severity of the conditions within different genotype and diagnosis, a stratified population was required. In these stratified patients, hyperthermia, a canonical sepsis indicator, could be a potential determinant of such a violation. In particular, sepsis patients were at higher risk of hyperthermia: carriers of the minor A allele developed around one more Celsius grade of temperature when stratified for final diagnosis and severity of the condition. At a lesser extent, this was also observed in non-infective SIRS. In conclusion, A allele carriers are prone to developing a SIRS criteria such as hyperthermia, but, at the same time, are protected from SIRS condition and, particularly, from sepsis. This hypothesis is corroborated by the linkage disequilibrium of rs11225395 with another MMP-8 genetic variant (rs1940475, lying less than 1 Kb apart from rs11225395), which has been investigated by infusion of LPS. The pattern (timing and levels) of cytokines and inflammation response was associated with rs1940475 [39] and this adds to the possibility of a genetic regulation of a classical or smoldering inflammatory picture. Being the A “proinflammatory” allele less represented in the septic group, but more prone to develop high body temperature, it should be postulated that fever is a continuously working mechanism for clearing bacteria (and putatively other DAMPS). While the relationship between temperature-related clearance of bacteria by neutrophils has already been observed experimentally [40,41], the relationship between fever and MMP-8 is reported for the first time. Possible pathways involved in this regulation could be EGFR [42] or bradykinin [43], both proteins capable to modulate MMP-8 activity. Patients able to develop moderate hyperthermia might therefore be protected from the development of sepsis. Consequently, it can also be extrapolated that infections/sepsis by pathogens occur at a higher than expected rate and local MMP8-mediated inflammation prevents spreading of the germ and pathogenicity.

### 4.2. MMP-1 and Intracellular Pathogens

A second result of the present study is the association between sepsis caused by intracellular pathogens and MMP-1 rs1799750 G insertion. In MMP-1, the insertion of a G base at −1609 creates an *ets* (erythroblast transformation specific) binding site that increases by around eight times the MMP-1 mRNA expression under inflammatory stimuli [14]. The apparent conclusion is that MMP-1 helps the pathogenicity or susceptibility of intracellular pathogens (i.e., Chlamydia, Mycoplasma, and Legionella). Further analysis is required to explain the molecular mechanisms for such an association.

If MMP-1 triggers susceptibility to intracellular pathogens, a possible mechanism could be the degradation of an (intracellular) antagonist or the enhancement of growth and diffusion of the microorganisms, putatively through remodeling of the extracellular matrix. In line with this hypothesis is that a protease-rich environment enhances the invasive properties of Mycoplasma Hyorhinis [44]. Under a molecular point of view, Mycoplasma triggers NF-KappaB (NF-kB) pathway activation [45] and *ets* has a coregulatory role in MMP-1 expression [46,47]. NF-kB stimulation has also been observed for Chlamydia [48] and Legionella [49]; in the latter study, moreover, a direct association between in vitro NF-kB expression and in vivo pathogenicity of the different strains could be demonstrated.

An alternative hypothesis is that, in a condition with more than 50% of patients without identification of a causative agent, MMP-1 could modify some preanalytical or analytical variants, leading to higher prevalence of identification in carriers of the variant accounting for higher expression. Since the diagnosis of these pathogens is based on three completely different techniques (namely, antigen immunofluorescence identification in urine, PCR based genetic material amplification, or antibody title) and none of them is singularly statistically associated to rs1799750 G insertion, this possibility should receive limited credit.

In conclusion, pathogen-related activation of the NF-kB pathway and MMP-1 rs1799750 G insertion, leading to *ets* binding in the promoter, could enhance the susceptibility of some patients to intracellular pathogens. Further investigation is required to demonstrate the mechanisms of MMP-1-related susceptibility. The present study suggests that triggering of *ets*/NF-kB-regulated protease(s) from a class of pathogens due to a limited number of genes might enhance intracellular pathogen invasiveness. Possible outcomes could be the development of novel therapeutic strategies (selective inhibitors of MMP-1 or the development of vaccines). Under the speculative point of view, the present results challenge the hypothesis that immune genetic variants can work independently of the pathogen causing sepsis.

### 4.3. MMP-3 and Virus Sepsis

The last original observation was the doubled risk of viral sepsis in the 5A carrier of MMP-3 rs3025058 -/* genotype. Under specific inflammatory conditions, the MMP-3 rs3025058 5A allele (i.e., deletion of A at position −1171) binds one or more nuclear stimulatory proteins (putatively NF-kB and Zinc-binding protein-89), thus increasing gene expression by two to three times [50]. Accordingly, within the human liver infected with HCV, the difference in MMP-3 expression between the 5A and 6A genotype is around ten times [51]. In contrast, the herniated intervertebral disk shows higher expression in 6A carriers [52]. Regardless of the real picture of the expression, in our study, MMP-3 genotype 6A was associated with a higher prevalence of viral sepsis, without a prevalent effect of a virus type over the others. Such a genetic variant has not been previously investigated in viral sepsis, while MMP-3 rs522616 (<1 Kb apart from rs3025058) was associated with severity of respiratory syncytial virus infection, but not to susceptibility, with MMP-3 plasma levels doubled in hospitalized cases compared to mild conditions treated at home [53]. It is not clear, thus far, whether MMP-3 expression during virus infection improves its virulence, assists in the development of a chronic disease, or represents a defensive mechanism of the host. Zta, an Epstein Barr virus protein, induces a four-time increase in MMP-3 in vitro expression which, in turn, is essential for cell invasion of the virus [54], although, on the other hand, MMP-3 exerts an indirect antiviral effect in macrophages infected with Dengue virus [6]. In this last study, MMP-3 translocates into the nucleus where it activates the NF-κB p65 subunit, enhancing the antiviral immune response. NF-κB itself enhances the gene expression of MMP-3 [6]. This loop could be further enhanced by functional genetic variants [50] such as rs3025058. At present, therefore, we report the association of virus sepsis diagnosis with a functional genetic variant of MMP-3. Whether this variant also influences, beyond susceptibility, prognosis is still unknown: we can only report a non-significant better prognosis of 5A (-/*) carriers (100% survival vs. 77.8 in A/A, *p* = 0.147, data not shown), but the reduced size of this sample does not allow firm conclusions. Further studies focusing on susceptibility and prognosis and MMP-3 variants hold great potential for risk stratification and treatment of many viral conditions, COVID-19 being the most renowned to date [55].

Finally, the possibility that MMP-3 variants modify pre-analytical conditions influencing the results of the microbiological test but not susceptibility, needs to be discussed. Present technology in the diagnosis of virus infection is indirect (i.e., based on IgM or high titer IgG antibody levels). Such a technique is notoriously resistant to pre-analytical variables, so a genuine biological association of functional genetic variant of MMP3 with virus infection can be bona fide demonstrated.

### 4.4. Comments and Limitations

Among the negative results, the lack of association with the diagnosis of bacterial sepsis needs further speculation. Two hypothesis can be put forward: the first is that some organs can have a different MMP expression. Several studies have shown that MMP genes are indeed upregulated during infection: different Gram-negative bacteria and lipopolysaccharide induce the transcription of MMP genes [56,57] and for the latter, this happens according to an organ- and time-specific mode [58]. Alternatively, a repertoire of the host’s proteinases is required only when the pathogen does not have proteases by itself [59].

Other studies have identified other MMP variants as associated with sepsis susceptibility or prognosis: MMP-1, MMP-3, MMP-8, and TIMP-1 variants have already been investigated in sepsis outcome in a few hundred ICU patients with severe sepsis, [10,22,37]. We cannot confirm the associations found in these previous studies: the possible reasons for such a discrepancy could be inclusion criteria, different age of patients, and setting of the studies (ICU vs. ICU plus medical ward). Even when analyzed by the specific class of pathogens causing the sepsis or gender, this association has not been identified (data not shown). A distinguishing factor for the present study is that it includes around one thousand patients and, for certain variants, is the largest study ever performed in sepsis. Study limitations can be found in the lack of plasma levels of the investigated proteases or TIMP-1, and in the limited number of genetic variants considered. Additionally, inclusion criteria in force at the time of the study ruled out of the investigation of patients with less than two SIRS criteria. Inclusion of patients with broader criteria would have provided a wider perspective on sepsis susceptibility and interaction with MMPs/TIMP-1 as well as with the Hardy–Weinberg equilibrium.

## 5. Conclusions

Our study challenges the concept of sepsis as a unique nosological entity, at least when investigating the role of MMP polymorphisms. While the MMP-8 functional variant could influence the inflammatory (innate immunity) response of the host, a role for single MMP variants can be spotted only for simpler pathogens, requiring a contribution of the host to acquire invasive properties through MMP expression. Thus, while bacteria have no association with any of the investigated MMP polymorphisms, intracellular pathogens, viruses, and fungus likely trigger MMP expression from the host. Genetic variants in loci of MMP-1 or -3 promoters binding NF-kB or *ets* transcription factors could therefore increase susceptibility to sepsis.

## Figures and Tables

**Table 1:** Demographic, clinical presentation, comorbidities, and clinical severity of the patients, according to the final diagnosis. SIRS criteria and comorbidities are described as positive/negative finding for the condition, while continuous variables are the median and, in parentheses, interquartile range. *p* = *p* values of Kruskal–Wallis or χ2 square test, WBC = white blood cells.

	Ni-SIRS (127)	Sepsis (812)	Debatable (119)	*p*
Male/female	66/61	426/386	52/67	0.201
Age	72 (59–84)	81 (72–87)	78 (71–87)	0.000
SIRS criteria				
Hyperthermia	20/107	389/423	33/86	0.0000
Hypothermia	4/123	33/779	3/116	0.658
Leukocytosis	55/72	459/353	77/42	0.002
Leukopenia	4/123	30/782	7/112	0.464
Tachycardia	112/15	608/204	95/24	0.003
Tachypnea	98/29	615/197	81/38	0.165
Immature leukocytes	0/127	3/809	0/119	0.634
Clinical presentation				
Breathing rate	22 (20–26)	24 (20–27)	22 (20–26)	0.301
Heart rate,	103 (96–110)	100 (90–110)	100 (90–108)	0.001
Temperature	36.5 (36–37.1)	37.6 (36.5–38.2)	36.7 (36–37.8)	0.000
WBC count	10.8(7.5–14.5)	12.8(9.2–16.7)	13 (9.4–15.8)	0.001
Comorbidities, severity and scores				
Charlson comorbidity index	2 (1–5)	3 (1–5)	3 (2–5)	0.008
SOFA Score	2 (1–3)	3 (2–4)	3 (2–4)	0.000
Apache II Score	10 (7–12)	12 (9–15)	11 (9–14)	0.000
SAPS Score	36 (27–40)	37 (34–43)	36 (34–42)	0.000

**Table 2:** Clinical presentation of sepsis and MMP8 rs11225935 (unstandardized). Comparisons were carried out with χ^2^ test or Mann–Whitney, according to the variable considered, and *p* values are reported on the right columns. *p* values legend: Ni-SIRS and Seps = comparison of SIRS criteria according to MMP-8 genotypes within each group (Ni-SIRS and Sepsis, respectively), GG and A* = comparison of SIRS criteria within a specific genotype (GG or A carrier) across the different groups of patients.

	Ni-SIRS		Sepsis		*p* values			
	GG (*n* = 33)	A* (*n* = 94)	GG (*n* = 289)	A* (*n* = 520)	Ni-SIRS	Seps	GG	A*
Hyperthermia	5/28	15/79	124/165	262/258	0.913	0.041	0.002	0.000
Hypothermia	1/32	3/91	13/276	20/500	0.964	0.653	0.695	0.758
Temperature	36.5 (36–37)	36.5 (36–37)	37.5 (36.5–38)	37.7 (36.6–38)	0.981	0.247	0.000	0.000
Leukocytosis	13/20	42/52	165/124	293/227	0.598	0.837	0.053	0.037
Leukopenia	1/32	3/91	10/279	19/501	0.964	0.887	0.898	0.824
WBC count	10.9 (7.9–13.6)	10.7 (7.2–14.9)	12.7 (8.9–16.7)	12.9 (9.4–16.8)	0.606	0.677	0.19	0.002
Tachycardia	30/3	82/12	229/60	377/143	0.574	0.034	0.109	0.002
Heart rate	107 (99.113)	101 (96–110)	100 (92–110)	100 (90–110)	0.359	0.576	0.017	0.011
Tachypnea	26/7	72/22	227/62	387/133	0.796	0.189	0.974	0.655
Breath rate	22 (20–26)	22 (20–26)	24 (20–26)	24 (20–26)	0.408	0.854	0.617	0.33
Immat. WBC	0	0	2/287	1/519	n.a.	0.262	0.632	0.67

**Table 3:** Genetic variants and groups of pathogens. Legend: a = codominant (− vs. −G vs. GG), *p* = 0.004. b = dominant (−/−G vs. GG) H.R. 6.2 95% C.I. 1.5–25.3, *p* = 0.004. c = codominant (− vs. −G vs. GG) *p* = 0.004. d = dominant (−/−G vs. GG), H.R. 7.7 95%CI 1.5–40.4, *p* = 0.005. e = dominant (−/−A vs. AA), H.R. 0.47 95% CI 0.23-.94, *p* = 0.032. f = T vs. Non-T H.R. 0.21, 95% C.I. 0.04–1.1 *p* = 0.041.

	MMP-1	MMP-3	MMP-8	MMP-9	TIMP-1
	rs1799750	rs3025058	rs11225395	rs2234681	rs4898
	−/−G/GG	−/−A/AA	AA/AG/GG	SS/SL/LL	Non-T/T
All	106/214/107	123/194/113	48/230/150	179/187/62	148/279
Gram+	37/73/30	46/59/35	15/75/50	51/67/22	48/92
Gram−	59/121/54	66/114/57	28/129/78	104/102/29	83/152
Intracell	3/0/6 a b	1/4/4	1/5/3	4/2/3	1/8
Virus	6/19/12	9/13/15 e	4/18/15	15/14/8	11/25
Fungus	1/1/5 c d	1/4/2	0/3/4	5/2/0	5/2 f
*p*	0.004	0.346	0.942	0.239	0.152

**Table 4:** Results of the last step of binary logistic regression for the inference of variables associated with all sepsis or sepsis caused by viruses or intracellular atypical bacteria.

	B Value (E.S).	Wald	Sig.	Exp(B)	95% CI EXP(B)	
**All sepsis**						
TIMP-1 rs4898 T carrier	−0.590 (0.265)	4.937	0.026	0.555	0.330	0.933
SOFA Score	0.227 (0.066)	11.908	0.001	1.255	1.103	1.427
Age	0.045 (0.007)	38.445	0.000	1.046	1.031	1.061
Hyperthermia	2.079 (0.315)	43.496	0.000	7.997	4.311	14.834
C Reactive Protein	0.013 (0.002)	41.767	0.000	1.014	1.009	1.018
MMP-8 11,225,395 Dominant	−0.720 (0.254)	8.032	0.005	0.487	0.296	0.801
Antimicrobial at home	0.706 (0.306)	5.317	0.021	2.025	1.112	3.690
Constant	−2.182 (0.706)	9.563	0.002	0.113		
**Virus**						
MMP-3 rs3025058 5A carrier	−0.838 (0.390)	4.613	0.032	0.433	0.201	0.929
COPD	1.086 (0.401)	7.352	0.007	2.963	1.351	6.498
Moderate/severe kidney disease	−2.467 (1.034)	5.692	0.017	0.085	0.011	0.644
Constant	−1.779 (0.325)	29.905	0.000	0.169		
**Intracellular atypical bacteria**						
TIMP-1 rs4898 C carrier	2.362 (0.906)	6.800	0.009	10.617	1.798	62.681
Previous myocardial infarction	2.263 (0.816)	7.699	0.006	9.613	1.943	47.545
Dementia	−2.607 (1.247)	4.373	0.037	0.074	0.006	0.849
MMP-1 rs1799750	2.638 (0.842)	9.819	0.002	13.982	2.686	72.795
Constant	−9.255 (1.985)	21.736	0.000	0.000		

## Data Availability

The data presented in this study are available on request from the corresponding author. The data are not publicly available due to privacy reasons.

## Supplementary Materials

The following supporting information can be downloaded at: https://www.mdpi.com/article/10.3390/biom12020279/s1, Figure S1: Flowchart diagram of the study, Table S1: Cross tabulation of MMP genetic variables and diagnosis, Table S2: Clinical presentation of sepsis and MMP8 rs11225935 in groups standardized for age and severity (SOFA, Charlson, Apache II, and SAPS score), Table S3: Association of MMP polymorphisms with severity and diagnosis of sepsis, Table S4: Association of MMP/TIMP polymorphisms with comorbidity, and severity scores, Table S5: Microorganisms identified in the population of sepsis patients.
