# Supplementary material for: Genetic Variants of Matrix Metalloproteinase and Sepsis: The Need Speed Study
Source: Biomolecules. 2022 Feb 9;12(2):279. doi: 10.3390/biom12020279 (PMC8961575; doi:10.3390/biom12020279)
Supplement: Supplementary file 1 [file biomolecules-12-00279-s001.zip › biomolecules-1562834-supplementary.pdf]

Figure S1. Flowchart diagram of the study.

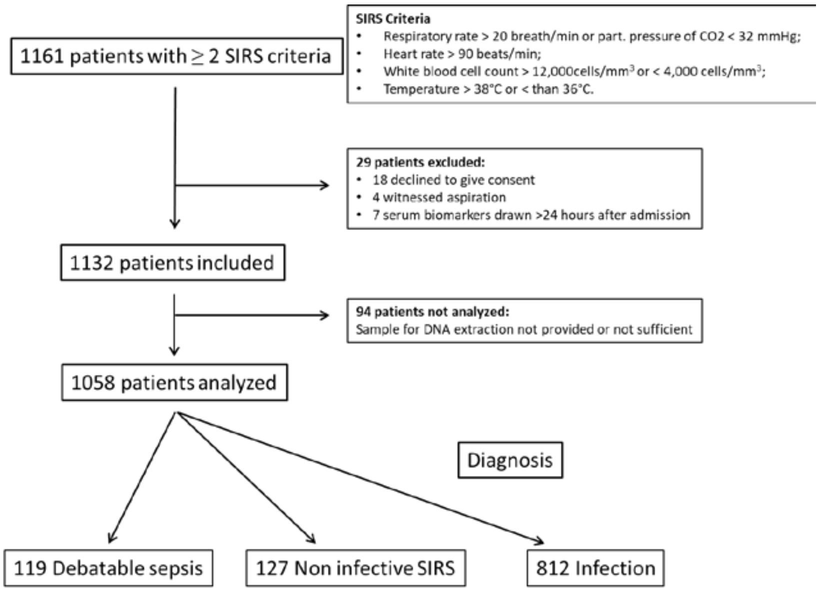

Table S1. Cross tabulation of MMP genetic variables and diagnosis. * in female patients only HW= Hardy Weinberh equilibrium, (all/sep/sirs)= P value of Hardy Weinberg equilibrium test in the whole cohort /sepsis/Ni-Sirs patients .

	**MMP1 rs1799750**	**MMP3 rs3025058**	**MMP8 rs11225395**	**MMP9 rs2234681**	**TIMP1 rs4898**
**Genotype**	**- -/-G/GG**	**--/-A/AA**	**GG/GA/AA**	**S S/S L/L L**	**Non-T/T**
**ALL patients**	**258/526/268**	**251/509/291**	**369/563/123**	**445/465/145**	**333/717**
Ni-SIRS	37/62/28	37/53/36	33/76/18	54/51/22	31/95
Sepsis	195/403/208	188/396/224	289/423/97	344/356/109	263/542
Debatable	26/61/32	26/60/31	47/64/8	47/58/14	39/80
HW (all/sep/sirs)	.922/.994/.833	.256/.612/.075	.00002/.002/.016	.18/.282/.128	.695/.827/.107*
Sepsis Vs Ni-SIRS (P values)					
Codominant	.423	.246	.099	.464	.07
Recessive	.697	.786	.032	1	
Dominant	.231	.125	.485	.245	
Overdominance	,805	.12	.113	.0714	
◎ 2			4.613	.416	3.282
**HR**			0.64 (0.42-0.97)	1.17 (.8-1.71)	.67 (.44-1.04)

Table S2. Clinical presentation of sepsis and MMP8 rs11225935 in groups standardized for age and severity (SOFA, Charlson, Apache II and SAPS score). Comparisons have been carried out with ◎ 2 test or Mann Withney, according to the variable considered, and P values are reported on the right columns. P values legend: N/S =comparison of SIRS criteria between Ni-SIRS and Sepsis patients, NiSIR and Seps = comparison of criteria according to MMP8 genotypes within each group (Ni-Sirs and Sepsis, respectively), GG and A* = comparison of criteria within a specific genotype (GG or A carrier) across the different groups of patients, WBC= white blood cells, Immat WBC= immature white blood cells.

	**Ni-SIRS (31)**		**Sepsis (60)**		**N/S**	**Ni-Sirs**	**Seps**	**GG**	**A***
**Rs 11225395**	**GG (9)**	**A* (22)**	**GG (23)**	**A* (37)**	**.379.**				
Hyperthermia	0/9	7/15	8/15	28/9	.001	.054	.002	.041	.001
Hypothermia	1/8	1/21	1/22	0/37	.226	.499	.201	.477	.191
Temperature	36.4(36-36.9)	36.9(36-38)	37.2(36.5-38.1)	38 (38-38.7)	.025	.185	.018	.03	.000
Leukocytosis	6/3	9/13	10/13	21/16	.767	.193	.317	.238	.239
Leukopenia	0/9	0/22	0/23	2/35	.304	n.a.	.257	n.a.	.267
WBC count	13.4(10.9-14.2)	10.6(7.1-17.9)	10.4(6.7-13.7)	12.3(8-14.7)	.839	.564	.523	.281	.987
Tachycardia	8/1	18/6	22/1	28/9	.948	.627	.044	.477	.582
Heart rate,	101(95-110)	105(97-110)	100 (95-118)	100 (90-107)	.338	.765	.133	.808	.212
Tachypnea	6/3	19/3	17/6	24/13	.212	.208	.464	.682	.072
Breath rate	19(16-21)	22(22-26)	24(20-27)	20(19-24)	.869	.008	.049	.02	.017
Immat WBC	0	0	0	0	n.a.	n.a.	n.a.	n.a.	n.a.

Table S3. Association of MMP polymorphisms with severity and diagnosis of sepsis. Inheritance reports the P values of ◎ 2 test according to the genotype. Legend: HW (micro/clinic) = P value of Hardy Weinberg equilibrium test in positive serology or microorganism growth/ clinically proven sepsis, HR Hazard Ratio.

	**MMP1**	**MMP3**	**MMP8**	**MMP9**	**TIMP1**
	**rs1799750**	**rs3025058**	**rs11225395**	**rs2234681**	**rs4898**
	**- -/-G/GG**	**--/-A/AA**	**GG /AG/AA**	**S S/S L/L L**	**Non-T/T**
Sepsis	195/403/208	188/396/224	289/423/97	344/356/109	263/542
		**Severity and outcome**			
Septic shock	16/26/12	13/23/18	22/27/5	15/31/8	15/39
30 days mortality	33/84/42	35/72/52	51/90/18	62/74/23	45/112
		**Diagnosis**			
Microbiologically proven	85/173/85	92/155/97	124/182/38	144/152/48	115/228
Clinical diagnosis	110/230/123	96/241/127	165/241/59	200/201/61	148/314
HW (micro/clinic)		.067/.401			
Blood culture positive	35/72/38	38/65/43	50/80/16	60/69/17	46/100
Blood culture negative	49/101/47	53/90/54	74/101/22	83/84/30	69/127
		**Inheritance**			
Codominant	.839	.079	.776	.919	.655
Dominant	.737	.044	.477	.744	
Recessive	.567	.757	.869	.731	
Sovradominant	.831	.049	.761	.929	
◎ 2		3.99/3.863			
**HR (95% CI)**		1.4(1.01-1.9)<br>1.32(1.001-1.8)			

Table S4. Comorbidity and Severity of condition according to the MMP/TIMP-1 genotype. All comparisons in c2 analysis yielded a P value > .05

	MMP1	MMP3	MMP8
	rs1799750	rs3025058	rs11225395
	- -/-G/GG	--/-A/AA	GG /AG/AA
Charlson.C.I.	3(1-5)/2(1-4)/2(1-5)	3(1-4)/2(1-4)/3(1-5)	3(1-5)/3(1-5)/2(1-4)
APACHE II	12(9-15)/12(9-14)/12(9-15)	11(9-14)/12(10-15)/12(8-15)	12(9-15)/12(9-15)/12(9-15)
SAPS Score	36 (30-41)/36(30-40)/35(30-42)	36 (30-41)/36 (30-41)/36 (30-42)	36(30-40)/36 (30-41)/36 (30-41)
SOFA Score	3(1-4)/2(1-4)/2(1-4)	2(1-4)/2(1-4)/3(1-4)	3(1-4)/2(1-4)/2(1-4)

	MMP9	TIMP1
	rs2234681	rs4898
	S S/S L/L L	Non-T/T
Charlson.C.I.	3(1-5)/2(1-5)/3(1-4)	3(1-5)/2(1-4)
APACHE II	12(9-14)/12 (9-15)/13(10-16)	12(9-15)/11(9-15)
SAPS Score	36 (30-41)/36 (30-41)/36 (30-41)	36(30-41)/36(30-41)
SOFA Score	2(1-4)/3(1-4)/2(1-4)	3(1-4)/2(1-4))

Table S5. Microorganisms identified in the population of sepsis patients. Associations between MMP polymorphisms and direct microbiological or serological diagnosis of sepsis are annotated. In detail: * P<.05 MMP-1 rs1799750 and Klebsiella Pneumoniae, MMP9 rs2234681 and Respiratory Syncytial Virus, MMP8rs11225395 and Clostridium difficile (0.029), and C carrier in TIMP1rs4898 with Enterococcus fecalis, MRSA, other Gram-positive bacteria and other viruses.

**Gram positive**	**140**	**Gram negative**	**237**
MSSA	28	Escherichia coli	113
Other streptococci	26	Pseudomonas aeruginosa	32
Enterococcus faecalis *	24	Proteus group	22
MRSA *	22	Klebsiella pneumoniae *	19
Clostridium difficile *	14	Other Gram negative	17
Other G positive *	10	Haemophilus influenzae	6
Streptococcus pneumoniae	9	Klebsiella oxytoca	4
Enterococcus faecium	7	Acinetobacter sp	3
**Viruses**	37	Enterobacter spp	7
Influenza A	16	Citrobacter sp	6
Other virus	6	Other enterobacteriacee	4
Respiratory Syncytial Virus	5	Serratia marcescens	2
Influenza B	4	Stenotrophomonas maltophilia	2
Adenovirus	3	**Atypical intracellular**	9
Parainfluenza	3	Mycoplasma pneumoniae	5
**Fungi and others**	7	Clamidia pneumoniae	2
Candida	4	Legionella pneumophila	2
Pneumocystis jirovecii	2		
Non TBC mycobacteria	1		
Tot 430			
